# Turning Fruit Seed Oils into High-Performance Open-Cell Polyurethane Foams: A Green Route to Petrochemical Polyol-Free Insulation

**DOI:** 10.3390/ma18235387

**Published:** 2025-11-29

**Authors:** Maria Kurańska, Elżbieta Malewska, Mateusz Barczewski, Joanna Banaś, Aleksandra Put, Julia Sędzimir, Hubert Ożóg, Natalia Kowalik, Marcin Zemła, Michał Kucała

**Affiliations:** 1 Faculty of Chemical Engineering and Technology, Cracow University of Technology, Warszawska 24, 31-155 Cracow, Poland; elzbieta.malewska@pk.edu.pl (E.M.); aleksandra.put@student.pk.edu.pl (A.P.); julia.sedzimir@student.pk.edu.pl (J.S.); hubert.ozog@student.pk.edu.pl (H.O.); natalia.kowalik18@student.pk.edu.pl (N.K.); marcin.zemla@doktorant.pk.edu.pl (M.Z.); 2 Institute of Materials Technology, Poznan University of Technology, Piotrowo 3, 61-138 Poznan, Poland; mateusz.barczewski@put.poznan.pl; 3 Faculty of Food Technology, University of Agriculture in Krakow, ul. Balicka 122, 30-149 Krakow, Poland; joanna.banas@urk.edu.pl; 4 Faculty of Chemical Engineering and Technology, CUT Doctoral School, Cracow University of Technology, Warszawska 24, 31-155 Cracow, Poland; michal.kucala@doktorant.pk.edu.pl

**Keywords:** fruit seed oils, biopolyols, open-cell foams, biopolyurethanes

## Abstract

**Highlights:**

**What are the main findings?**

- Fruit seeds can serve as a source of raw materials for the synthesis of polyurethanes.
- Open-cell polyurethane foams can be produced without the use of petrochemical polyols.

**What are the implications of the main findings?**

- The viscosity of oils has a significant impact on the properties of the resulting foams.
- Transesterification of fruit seeds enables the production of reactive hydroxyl derivatives suitable for foam synthesis.

**Abstract:**

Five types of fruit seed oils have been described from the perspective of their potential use in the synthesis of biopolyols. The overall goal is to increase the participation of biopolyurethanes in polymer production, aligning with the European Green Deal. Blackcurrant, cherry, grape, pomegranate, and watermelon seed oils were characterized by iodine value, acid value, density, average molecular weight, viscosity, and fatty acid profile. The thermal properties of the oils were also determined using thermogravimetry (TGA) and differential scanning calorimetry (DSC). In order to obtain reactive compounds for the synthesis of biopolyols, the vegetable oils were modified using the transesterification reaction with triethanolamine. The resulting biopolyols were characterized by their hydroxyl number, acid number, density, average molar mass, and viscosity. The biopolyols were then used to produce thermal-insulating polyurethane foams by completely replacing petrochemical polyols with counterparts derived from fruit seeds. The obtained foams were described by their closed cell content, apparent density, thermal conductivity coefficient, dimensional stability, maximum stress at 10% deformation, thermal stability, oxygen index, and water absorption. In addition, an analysis of the foaming process revealed that the properties of fruit seed oil after chemical modification had an impact on the properties of the open-cell polyurethane foams and the foaming process itself.

## 1. Introduction

The European Green Deal was established in 2019 to make Europe the first climate-neutral continent by 2050 and accelerate the transition to a circular economy model. It is described by the European Commission as the “bedrock” of the Ecodesign for Sustainable Products Regulation initiative. Together with the Circular Economy Action Plan comprising the Sustainable Products Initiative and the European Industrial Strategy, both announced in the European Green Deal and published together in March 2020, they form the core rationale for the Ecodesign for Sustainable Products Regulation proposal [1]. The Circular Economy Action Plan states that up to 80% of the environmental impacts of products are determined during the design phase.

Taking into account the latest regulations of the European Union, it is necessary to search for new raw materials for the synthesis of polymers and design their recycling to enable the possibility of recovering substrates. Polyurethanes are a widely used group of materials. They find applications in various fields, including medicine, the automotive industry, construction, and sports.

Polyols are one of the two main substrates for the synthesis of polyurethanes (PUR). PUR is produced using various methods and in many forms, offering a wide range of products. These can be foam materials, coatings, elastomers, adhesives, or impregnates [2]. On the global market, flexible and rigid polyurethane foams account for the largest share of PUR production. Flexible PUR foams are used, among other applications, in the furniture and automotive industries, while rigid PUR foams are used as excellent thermal insulation materials in construction and refrigeration. In 2023, PUR production accounted for 5.3% of the global production of polymer materials, equivalent to approximately 21.93 million tons. In Europe, PUR production amounted to 2.97 million tons (5.5% of total production) [3].

Due to the limited resources of petrochemical raw materials and their increasing prices, the search for new raw materials for the production of polymer materials is currently a key issue. Owing to their versatile properties, polyurethane materials are essential in many areas of industry and everyday life, which is why their sustainable development is particularly important. The use of renewable raw materials reduces the carbon footprint during production and limits the consumption of fossil raw materials [4]. Biopolyols for the synthesis of polyurethanes can be obtained using a wide range of raw materials, of which vegetable oils subjected to chemical modification are the most popular. The main chemical modification routes for vegetable oils are ozonolysis, epoxidation and opening oxirane rings, hydroformylation, transesterification, and transamidization reactions [5]. In the transesterification method, various chemical compounds can be used, including ethylene glycol, propylene glycol, diethylene glycol, glycerin, and triethanolamine [6]. The triethanolamine transesterification method enables a fast and efficient synthesis of biopolyols. Given the presence of tertiary amines, their products are characterized by high reactivity and can serve as substrates for the synthesis of polyurethanes. Such biopolyols are characterized by higher reactivity, as confirmed by studies on the foaming process [7]. In those studies, three different biopolyols and a petrochemical polyol were used. The presence of a tertiary amine built into the chemical structure of the polyol led to the highest reactivity of the PUR system. The use of biopolyols with higher reactivity has a significant impact on the preparation of open-cell polyurethane spray foams [8].

For the synthesis of biopolyols from vegetable oils, vegetable oils from the most common oil plants, such as rapeseed, soybean, palm or sunflower, are most extensively used [9,10,11,12,13,14,15]. Fruit seeds, which are waste or a by-product in fruit processing, may be a promising source of oils. Their use in the synthesis of biopolyols has not yet been widely studied, and the attempts undertaken so far have concerned only a few fruit species. Oils from blackcurrant, cherry, grape, pomegranate, and watermelon seeds are an attractive object of research in this respect.

Every year, thousands of tons of fruit seeds are discarded worldwide as by-products of the agri-food industry. Meanwhile, these seeds are characterized by a high content of oils rich in monounsaturated fatty acids. Fruit seeds can be a valuable raw material for various industries, potentially also for biopolyol producers. There are studies in the literature on the production of polyols from passion fruit seed oil and colloquia watermelon oil [16,17,18].

The largest fruit producers are India, Vietnam, and China. In 2017, global fruit production totaled 33.63 million tons. The most produced fruits are watermelons (118 million tons), bananas, apples, grapes, and oranges. In Poland, apple production is the dominant sector, amounting to 3.4 million tons in 2024. In the category of tree fruit (excluding apples), the largest harvests correspond to cherries (110 thousand tons). When it comes to berries, the production of strawberries (159 thousand tons) and currants (100 thousand tons, including 68 thousand tons of blackcurrants) is the greatest. Although most of them are consumed, fruits are a food group with a high level of losses, amounting to over 20% of global production. Losses are primarily due to the perishability of fruits, aging, rotting, or unsuitable appearance resulting from damage. Fruit pits and peels are also a by-product of processing. In the countries that are the leading producers, over 50 million tons of fruit waste and by-products were generated between 2007 and 2012 [18].

Food waste includes both waste and by-products of processing, as well as finished and unconsumed products. A significant portion of this waste ends up in landfills and rivers, negatively impacting the natural environment. In Poland, 90% of processing waste is sold or directed to composting plants and biogas plants [19,20]. An alternative use of fruit waste is to process it into feed for farm animals or value-added products, such as dietary supplements, pigments, biofuels, biopolymers, essential oils, and edible oils [21,22].

The variety of available oil sources drives the search for new raw materials for synthesizing components used in the production of polymers, in line with the eco-design of environmentally friendly biomaterials. In this study, five types of fruit seed oils were selected for biopolyol synthesis due to their availability as by-products of the fruit processing industry and thus their relevance as waste materials within the circular economy framework. The selected fruit seeds (blackcurrant, cherry, grape, pomegranate, and watermelon) represent significant waste streams generated annually in large quantities. Oils extracted from these seeds are characterized by a high content of unsaturated fatty acids, which enhances their reactivity in chemical modification processes leading to biopolyol formation. An additional selection criterion was the diverse fatty acid composition of these oils, allowing the assessment of how chemical structure influences the properties of the resulting biopolyols and polyurethane systems.

## 2. Materials and Methods

### 2.1. Materials for Biopolyol Synthesis

Blackcurrant seed (O_BC), cherry seed (O_CH), grape seed (O_GR), pomegranate seed (O_PG), and watermelon seed (O_WM) oils were purchased from Ol’Vita (Mysłaków, Poland). Triethanolamine was purchased from Avantor Performance Materials Poland (Gliwice, Poland). The purity level, according to the manufacturer, is ≥99%. Anhydrous zinc acetate from Chempur (Piekary Śląskie, Poland), with a purity of ≥97%, was used as the catalyst.

### 2.2. Materials for Open-Cell Foam Preparation

The polyol component consisted of biopolyols obtained by transesterification of fruit seed oils. The biopolyols were designated as follows: P_BC—biopolyol from blackcurrant seed oil, P_CH—biopolyol from cherry seed oil, P_GR—biopolyol from grape seed oil, P_PG—biopolyol from pomegranate seed oil, P_WM—biopolyol from watermelon seed oil. PMDI—polymeric diphenylmethylene 4,4’-diisocyanate, with a content of free isocyanate groups of 31% by mass, was used as the isocyanate (Purinova, Bydgoszcz, Poland). Amine catalysts (Polycat^®^ 15—bis [3(dimethylimino)propyl]amine, Polycat^®^ NP10) and an organometallic catalyst (Kosmos^®^ 19—dibutyltin dilaurate) were used to obtain open-cell PUR foams. The catalysts were supplied by Evonik Industries AG (Essen, Germany). The surfactants used were ORTEGOL^®^ 500, a polyether–polysiloxane copolymer, and TEGOSTAB^®^ B 8526, a polyether–polysiloxane copolymer supplied by Evonik Industries AG (Essen, Germany). In order to reduce the flammability of biomaterials, liquid flame retardant TCPP—tris(2-chloroisopropyl)phosphate with a viscosity of 67 mPa·s at 25 °C was employed. Water played the role of a chemical foaming agent.

### 2.3. Biopolyol Synthesis

Biopolyols from fruit seed oils were obtained by transesterification with triethanolamine (TEA). The reactions were carried out in three-necked flasks with a capacity of 2 dm^3^. The set consisted of a heating bowl, a three-necked flask, an air cooler, a thermocouple, and a mechanical stirrer. The molar ratio of TEA to oil was 3:1. Anhydrous zinc acetate was used as a catalyst in the amount of 0.3% by mass in relation to the total mass of oil and triethanolamine. The substrates were placed in a flask and then heated to 175 °C, with constant stirring. After reaching the target temperature, the reaction was carried out for 2 h. For each oil, three syntheses were carried out.

### 2.4. Open-Cell Polyurethane Biofoam Preparation

Biofoams were prepared using a one-step method from two components (A and B). Component A (polyol premix) consisted of a biopolyol, catalysts, flame retardant, blowing agent, and surfactants to open the cells and stabilize the structure of the foams (Table 1). Component B was isocyanate. First, the components of the polyol premix were mixed for 15 s, and then component B was added. The mixture was remixed for approximately 5 s. The systems prepared in this way were poured into an open mold, where growth occurred in a vertical direction. The final materials were conditioned for 24 h at 22 °C and 50% relative humidity before being cut and tested. Table 1 presents the formulations of the biopolyurethane foams. The amount of PMDI was selected appropriately for each formulation, considering the hydroxyl number of the biopolyol, to maintain an isocyanate index value of 1.1.

### 2.5. Testing Methods for Oils and Polyols

The fatty acid profile of the oils was determined using a GC-2010 Pro gas chromatograph (Shimadzu, Kyoto, Japan) equipped with a flame ionization detector (FID). The oils were previously transesterified with methanol according to ISO 5509 [23]. Individual fatty acid methyl esters were identified by comparison with the standard Supelco 37 FAME Mix (Sigma-Aldrich Co., St. Louis, MO, USA). Separations were performed on an SH-FAME Wax column (30.0 m × 0.32 mm × 0.25 m) with helium 5.0 (as carrier gas) at a flow rate of 30 mL/min. The column was heated at the following temperature settings: 60 °C for 2 min, then increased to 200 °C at a rate of 10 °C/min, followed by an increase to 240 °C at a rate of 5 °C/min and held at this temperature for 7 min. The injector and detector temperatures were set to 240 °C and 250 °C, respectively. Every sample was analyzed in triplicate.

Gel permeation chromatography (GPC) was used to determine the average molecular weights of oils and biopolyols. An Azura^®^ chromatograph from Knauer (Berlin, Germany) was equipped with thermostated columns and a refractometric detector. The analysis was performed at 35 °C using tetrahydrofuran as the eluent at a flow rate of 1 cm^3^/min. The number-average molar masses (Mn) and weight-average molar masses (Mw) were determined using calibration with standard polystyrene standards.

The determination of iodine value (I_V_) was performed using the Hanus method following the guidelines of the PN-87/C-04281 standard [24]. This procedure involves an addition of halogen atoms at the sites of double bonds in unsaturated compounds. The unsaturation degree of the compound is expressed as the mass of iodine that has been added. In this method, a sample of the analyte is weighed into a volumetric flask, and then an excess of iodine bromide (IBr) solution in glacial acetic acid is added. Depending on the degree of unsaturation of the tested substance, the time required for the addition of IBr ranges from 30 to 60 min. The excess of iodine bromide is determined using a potassium iodide (KI) solution, which reacts with IBr to form iodine (I_2_). The released free iodine (I_2_) is then titrated with an aqueous solution of sodium thiosulfate (Na_2_S_2_O_3_). A starch solution is used as an indicator. In the presence of iodine, it takes on a characteristic garnet color. The sample is titrated until the garnet color disappears completely. Iv was calculated according to the following formula:

$$Iv=\frac{\left(V_{1}-V_{2}\right)\cdot C_{Na_{2}S_{2}O_{3}}\cdot 0.1269}{m}\cdot 100\left[\frac{{gI}_{2}}{100g}\right]$$

where V_1_—volume of Na_2_S_2_O_3_ solution used for titration of the blank, cm^3^; V_2_—volume of Na_2_S_2_O_3_ solution used for titration of the sample, cm^3^; CNa_2_S_2_O_3—_concentration of Na_2_S_2_O_3_ solution, mol/dm^3^; 0.1269—number of grams of I_2_ corresponding to 1 mL of Na_2_S_2_O_3_ solution with a concentration of 1 mol/dm^3^; m—sample mass, g.

The determination of the hydroxyl number (OHv) of oils, biopolyols, and rebiopolyols was carried out using a pyridine-free method. The essence of the method lies in the acetylation reaction of hydroxyl groups using pyromellitic anhydride in acetone, catalyzed by 1-methylimidazole. Then, the excess anhydride is decomposed using water. The resulting acid is titrated with a NaOH solution in the presence of an indicator—a solution of thymolphthalein in ethanol. OHv was calculated according to the formula

$$OHv=\frac{\left(V_{1}-V_{2}\right)\cdot C\cdot 56.11}{m}\left[\frac{mgKOH}{g}\right]$$

where V_1_—volume of NaOH solution used for titration of the blank, cm^3^; V_2_—volume of NaOH solution used for titration of the sample, cm^3^; C—concentration of NaOH solution, mol/dm^3^; 56.1—molar mass of KOH, g/mol; m—sample mass, g.

The functionality of biocomponents was calculated according to the formula

$$f=\frac{M_{n}\cdot LOH}{56100}$$

where f—functionality, M_n_—number average molecular weight of biopolyol, g/mol, LOH—hydroxyl number, mgKOH/g; 56,100—number of milligrams of KOH corresponding to 1 mole of hydroxyl groups, mgKOH/mol.

The viscosity of oils and biopolyols was measured using a Lamy Rheology CP-4000 (Lamy Rheology Instruments, Champagne-au-Mont-d’Or, France) device via the plate–plate method. Measurements were taken at a temperature of 25 °C and a rotation speed of 100 rpm. The density of oils and biopolyols was determined using the areometric method. A Gomar (Warsaw, Poland) areometer with a measuring range of 0.9–1.0 g/cm^3^ was used. A 250 cm^3^ measuring cylinder was filled with the tested substance, and then the areometer was placed in it. After the time required for the device to stabilize in the liquid had elapsed, the result was read.

The water content was determined by the Karl Fischer method, as specified in PN-81/C-04959 [25], using a TitroLine TA 05 plus device from SCHOTT Instruments GmbH (Mainz, Germany). This method is based on the reaction of the Fischer reagent with water, and the equivalence point is determined by volumetric titration.

Chemical structure studies of oils and biocomponents were performed using a Nicolet iS5 FTIR spectrometer (Thermo Fisher Scientific, Waltham, MA, USA). The instrument was equipped with an ATR i7D attachment with a diamond crystal. Spectra were recorded in the infrared range of 4000–500 cm^−1^ in 24 scans.

The thermal properties of the materials were investigated by differential scanning calorimetry (DSC) using a DSC 204 F1 214 Polyma from Netzsch (Selb, Germany). Samples weighing 10 ± 0.2 mg were heated from −60 to 100 °C at a rate of 10 °C/min, held at this temperature for 10 min, and then cooled back to room temperature at a cooling rate of 10 °C/min. Measurements were performed in an inert gas atmosphere in gold-coated high-pressure crucibles. The thermal stability of oils and biopolyols was investigated by thermogravimetry (TG) in the temperature range of 0–700 °C using a TG 209 F1 device (Netzsch, Selb, Germany). Heating was carried out at a rate of 10 °C/min in an inert gas atmosphere. Samples weighing 10 mg ± 0.1 mg were placed in alumina (Al_2_O_3_) crucibles.

### 2.6. Testing Methods for PU Systems and Biofoams

The foaming process was analyzed using a FOAMAT^®^ apparatus manufactured by Format Messtechnik GmbH (Karlsruhe, Germany). The apparatus is equipped with a computer, a laboratory scale, a mechanical stirrer, an ultrasound sensor, a pressure gauge, and a thermocouple. During the foaming process, the following parameters are measured: temperature, pressure, dielectric polarization, mass, and foam height as a function of time. Samples are prepared by making a polyol premix and weighing the isocyanate, then mixing the components and pouring them into a measuring tube. The thermocouple is placed in a previously drilled hole in the tube, and the apparatus starts the measurement.

The morphology of cells was analyzed using a scanning electron microscope TM3000 (Hitachi, Tokyo, Japan). Foam samples of 1 × 1 × 1 cm before observation were covered with gold using a Polaron SC7640 duster (Quorum Technologies Ltd., Laughton, UK). The sputtering process was carried out for 90 s at a current of 10 mA. Observations were carried out at an accelerating voltage of 15 keV. The apparent density of the foam materials was determined in accordance with the ISO845:2006 standard [26]. The samples were measured and weighed to an accuracy of 0.01 mm and 0.01 g, respectively. Closed cell content was measured according to PN-ISO 4590 [27]. The contents of closed cells in those five samples (3 cm × 3 cm × 10 cm) were measured and averaged. Thermal conductivity was found following ISO 8301 [28] over a temperature gradient from 0 to 20 °C using a heat flow meter instrument Fox200 (TA Instruments, New Castle, DE, USA) and foam samples with dimensions of 5 cm × 20 cm × 20 cm. The thermal conductivity coefficients of three samples were measured and averaged. Measurements of compressive strength were carried out in accordance with the EN ISO 844:2021 standard [29]. The samples were tested in a direction parallel and perpendicular to the direction of foam growth. The compressive force was applied at a speed of 2 mm/s, axially in a perpendicular direction to the square surface. The compressive strength was measured at 10% deformation using a Zwick 1445 instrument (Zwick Roell Group, Ulm, Germany). The compressive strength of five samples (5 cm × 5 cm × 5 cm) was measured and averaged.

## 3. Results

Functionalization of vegetable oils can proceed by modifying ester bonds or double bonds. From the point of view of modifying double bonds, it is important to determine the iodine number, which determines the content of unsaturated bonds. In order to examine the suitability of fruit seed oils for the synthesis of biopolyols, their comprehensive characterization was carried out. Table 2 presents the profile of fatty acids used for the synthesis of biopolyols.

Linoleic acid is the dominant fatty acid in all the samples studied, with the largest share corresponding to polyunsaturated fatty acids (PUFAs). The content of linoleic acid in O_BC, O_CH, O_GR, O_PG, and O_WM oils is 54, 59, 77, 48, and 57, respectively. In the literature, the content of linoleic acid is reported to be in the range of 41–48%, 31–46%, 62–78%, 4–39%, and 53–71% [18]. The slight differences may be attributed to variations in plant species, cultivation area, and weather conditions. Considering the diversity of plants, it is reasonable to conduct comparative studies and analyze whether the differences in chemical structure have a significant impact on the properties of the biopolyols obtained from them and, consequently, whether these differences affect the properties of PUR foams. The highest content of saturated fatty acids (SFAs) among the oils studied is found in watermelon seed and pomegranate seed oils. In O_WM, these are mainly palmitic and stearic acids, while in O_PG, stearic and behenic acids. Monounsaturated fatty acids (MUFAs) constitute 7–24% of the acids in the oils studied.

**Figure 1:**
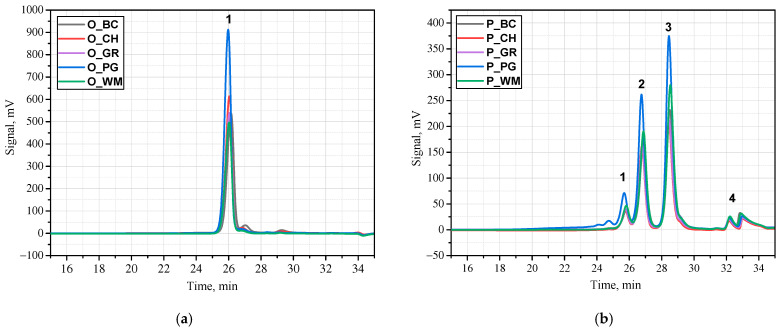
GPC chromatograms of oils (**a**) and biopolyols (**b**).

In the next stage of the research, the oils were characterized by determining their average molecular weights, dispersity, density, viscosity, and iodine value (Table 3). The same analysis was performed for the biopolyols obtained by transesterification of the appropriate oils with triethanolamine (Table 4). Figure 1 shows chromatograms of both the fruit seed oils (a) and the biopolyols (b) obtained from them.

The number-average molecular weights of the oils range from 770 to 881 g/mol. These are characteristic values for triglycerides of higher fatty acids. The highest average molecular weight has been found in pomegranate seed oil, which may be attributed to its high content of long-chain behenic acid. The oils are characterized by low dispersibility, with a value that does not exceed 1.05. The average molecular weights of the biopolyols obtained are similar, with Mn approximately 275 g/mol and Mw approximately 470 g/mol. An exception is the biopolyol from pomegranate seed oil (P_PG), whose average molecular weight is approximately one-third higher than that of the other biopolyols. The dispersibility of the molar masses of the biopolyols is about 1.7. The presence of individual components was confirmed by GPC measurements (Figure 1). It results from the formation of products of different chain lengths during transesterification, such as tri-, di- and monoglycerides, glycerol and unreacted triethanolamine and mono-, di- or trisubstituted triethanolamine derivatives. The GPC-based chromatograms showed characteristic peaks of triglycerides (peak 1), diglycerides (peak 2) and monoglycerides (peak 3). Peak 4 comes from unreacted amine or glycerine (Figure 1b).

The viscosities of the obtained biopolyols were approximately three times higher than those of the starting oils. This effect may be due to the lower branching of molecules and increased friction between them. The highest viscosity of 528 mPa∙s was measured for biopolyol P_PG. Taking into consideration the fact that the acceptable viscosity of industrial polyols should not exceed 5000 mPa∙s, it can be stated that the viscosities of the obtained biopolyols are favorable from the application point of view. The density of the oils tested was about 0.93 g/cm^3^. After the transesterification reaction, a slight increase in density of about 0.06 g/cm^3^ was observed. The iodine values found for grape seed and watermelon oils are similar to the literature values [18]. In the case of O_BC, O_CH, and O_PG, the results differ, which is due to the differences in fatty acid profiles resulting from the variety of agrotechnical conditions. The iodine values, as expected, follow the contents of unsaturated fatty acids. Oils with a higher content of MUFA and PUFA—blackcurrant, cherry, and grape seed oil—are characterized by the highest Ivs. Iodine values are lower for pomegranate and watermelon seed oils because they contain higher amounts of saturated acids.

The most crucial property of chemically modified vegetable oils is the hydroxyl number. Its value determines the amount of the isocyanate component and the properties of foams. The hydroxyl numbers of the obtained biopolyols were about 370 mgKOH/g. Polyols with such hydroxyl numbers can be successfully used for the synthesis of rigid polyurethane foams. Biopolyols described in the literature derived from rapeseed, sunflower and linseed oil synthesized by transesterification of TEA are characterized by similar OHv values in the range of 302–384 mgKOH/g [30,31].

Among the obtained biocomponents, the biopolyol derived from pomegranate seed oil exhibited the highest functionality, exceeding 2. The dispersity of the remaining biopolyols was about 1.8 (Table 4). The relatively low functionality likely results from the formation of products containing one or no hydroxyl groups per molecule during the modification process. However, this value is close to 2, which does not exclude the possibility of using such biocomponents in PUR synthesis. The functionalities of the biopolyols from rapeseed oil modified by transesterification with TEA are slightly higher and, depending on the molar ratio of oil to triethanolamine, in the range of 1.80–2.25 [32].

**Figure 2:**
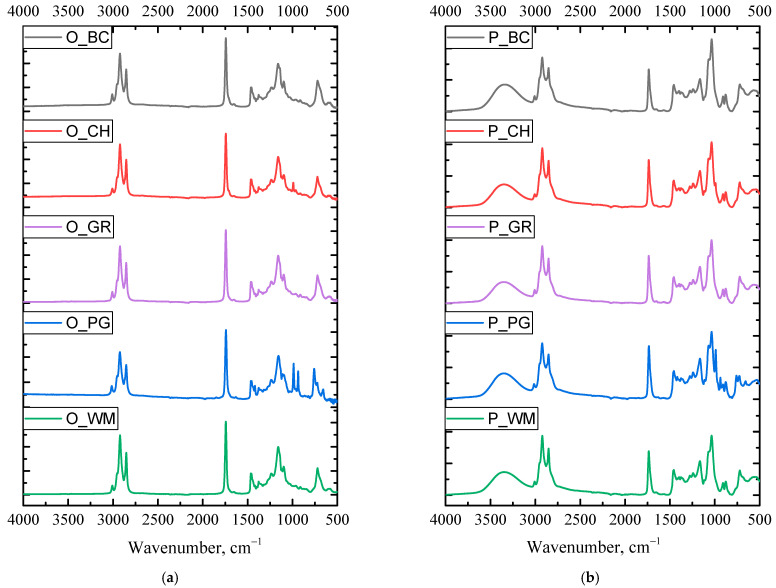
FTIR spectra of fruit seed oils (**a**) and biopolyols based on fruit seed oils (**b**).

The absorption spectra of the tested oils are presented in Figure 2.

The analysis of the FTIR spectra of the tested oils indicates that they are characterized by a similar chemical structure (Figure 2a). The presence of a strong band around 1700 cm^−1^ indicates the presence of a carbonyl group, while the band in the region of C-O stretching vibrations at 1300–1100 cm^−1^ reveals the presence of ester bonds. In the spectra of the obtained biopolyols (Figure 2b), a characteristic band of unbound hydroxyl groups can be observed in the region of 3340–3390 cm^−1^. Hydroxyl groups can also be recognized by a strong band in the range of C-O stretching vibrations at 1260–1000 cm^−1^ [33]. A weak band in the range of 1250–1020 cm^−1^ may originate from a non-conjugated C-N bond and suggest the presence of tertiary amines.

**Figure 3:**
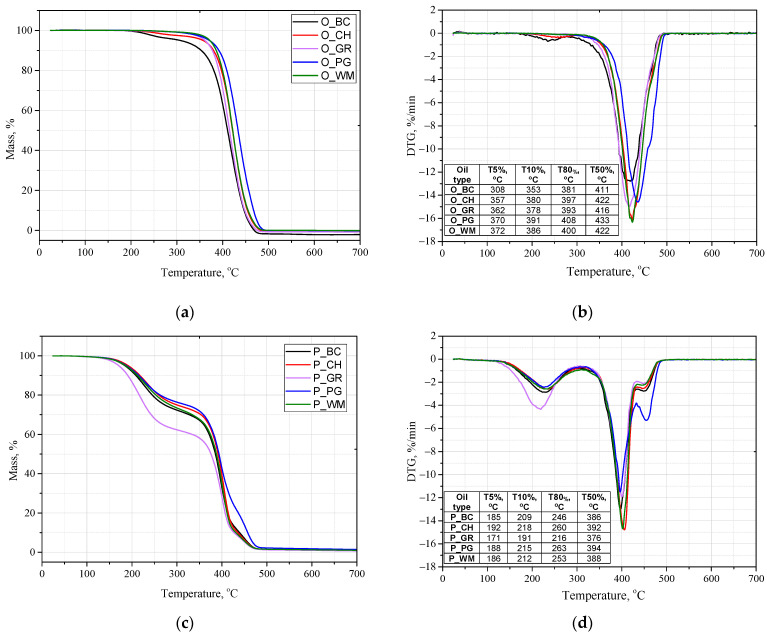
TG (**a**,**c**) and DTG (**b**,**d**) spectra of fruit seed oils and biopolyols based on these fruit seed oils.

**Figure 4:**
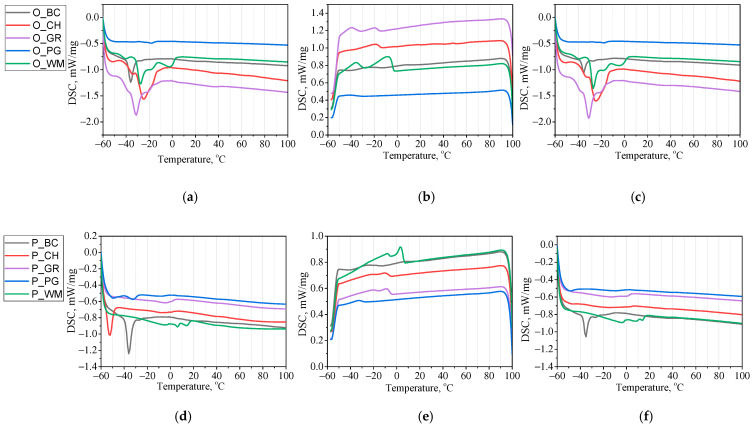
DSC curves: first heating of oils (**a**) and biopolyols (**d**); cooling of oils (**b**) and biopolyols (**e**); second heating of oils (**c**) and biopolyols (**f**).

The fruit seed oils and biopolyols obtained from them were also analyzed for thermal properties. The TG and DSC curves of the oils and biopolyols are presented in Figure 3 and Figure 4.

In the case of the oils, the 50% mass loss temperature values range from 411 °C (O_BC) to 433 °C (O_PG). The lowest T5% temperature, 308 °C, is characteristic of the blackcurrant seed oil. However, it exceeds the temperature of the transesterification reaction, which ensures the stability of the oil during the process. In the case of biopolyols, decomposition occurs in several stages due to the presence of various compounds in the mixture. The lowest T5% decomposition temperature is characteristic of biopolyol P_GR (171 °C), while the highest one was found for P_CH (192 °C). The T50% temperatures for the biopolyols ranged from 376 °C (P_GR) to 394 °C (P_PG). Oil O_PG is characterized by a considerable mass loss at around 450 °C. This effect may be related to its higher molecular weight. The range of decomposition temperatures of the obtained biopolyols is similar to that of the biopolyol obtained by epoxidation of linseed oil [34]. In the synthesis of PUR foams, due to the exothermic nature of the reaction, the temperature typically reaches approximately 160 °C. Therefore, the use of biopolyols from fruit seed oils for the synthesis should not result in their degradation.

The DSC analysis showed that both the oils and the biopolyols melt at temperatures below 0 °C. According to the DSC analyses conducted so far for edible oils (rapeseed oil, sunflower oil, olive oil), they melt at temperatures ranging from −40 °C to 0 °C. The melting temperature range is similar to the reported fruit seed oils [35]. During the heating process, a strong endothermic effect was observed for biopolyols P_BC and P_CH at temperatures of −36 °C and −52 °C, respectively. For the remaining biocomponents, the melting temperatures exceeded the measurement range. This means that they melt at temperatures lower than −60 °C. The cooling curves of the biopolyols reveal weak exothermic effects in the range from −32 °C to 4 °C, suggesting gradual crystallization. Due to the low melting and crystallization temperatures, the obtained biocomponents could potentially be used in a wide temperature range. The DSC curves of the oils and the biopolyols during the heating and cooling processes are shown in Figure 4.

**Figure 5:**
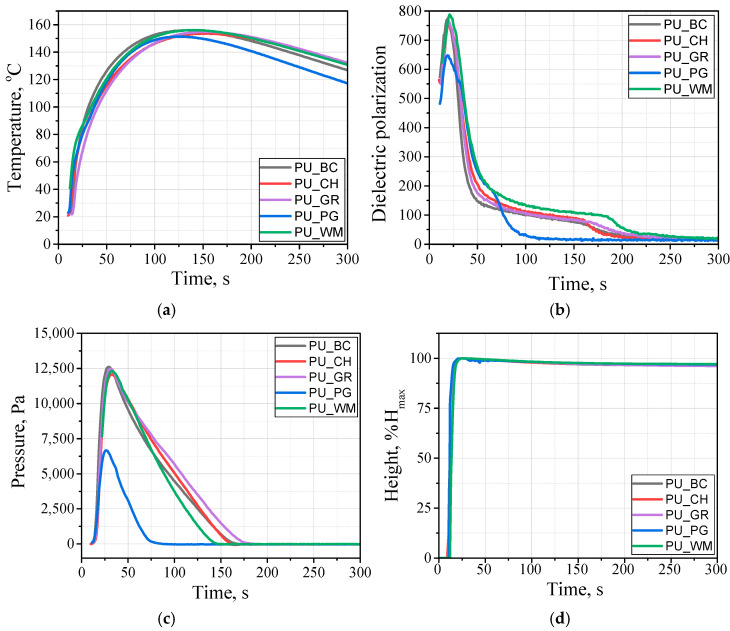
Influence of the type of biopolyol on changes in temperature (**a**), dielectric polarization (**b**), pressure (**c**), and height during the foaming process (**d**).

One of the most essential stages in the synthesis of porous polyurethane biomaterials is the foaming process. At this stage, a cellular structure is formed, which determines both thermal insulating and mechanical properties. Introducing new components into the formula requires a thorough analysis of the foaming process. Figure 5 shows the effect of the biopolyols from various fruit seed oils on the reactivity of polyurethane systems expressed by the dielectric polarization change curve and temperature changes during the foaming process.

Obtaining biocomponents from renewable raw materials is a crucial aspect of promoting green chemistry. However, demonstrating real application possibilities of such biocomponents is the basis for eco-designing environmentally friendly biomaterials. To meet the latest European Union regulations on limiting greenhouse gas emissions, open-cell polyurethane foam recipes for thermal insulation applications have been developed. These developed biomaterials are environmentally friendly for two reasons. Firstly, these are materials in which one of the two main components has been replaced with a component from renewable raw materials. Secondly, these are thermal insulation materials that reduce heat losses and thus polluting emissions associated with building heating.

The foaming process was characterized by a similar course regardless of the type of biopolyol. In the case of the PUR system modified with P_PG, a faster reduction in dielectric polarization in the matrix cross-linking phase may have resulted from the higher molecular weight of this biopolyol. Changes in dielectric polarization correlate with changes in the PU_PG pressure. In the PU_PG system, the pressure value is the lowest, and this is related to the faster cross-linking of the PUR matrix, which is why the expanding material does not exert pressure on the measuring table. Such a measurement, in a very good way, reflects the impact of the material during the foaming process on the mold walls. A short gelling time and a higher rise velocity favor the formation of foams at lower pressures [36].

**Figure 6:**
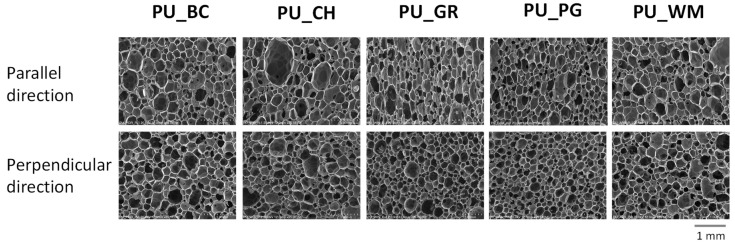
SEM microphotographs of biofoams based on modified fruit seed oils.

The foaming process has a significant impact on the cellular structure of foams. Foaming PUR systems in an open form affect the production of foams with anisotropic cell shapes, i.e., their shape differs depending on the direction of testing. SEM images show an elongated cell shape in the direction of material growth (Figure 6).

The surface perpendicular to growth is characterized by isotropic cells. This phenomenon has a direct effect on mechanical properties. The stress value at 10% compression in a direction parallel (σ pa) to the growth direction is characterized by higher values than the one measured in a perpendicular direction (σ pe).

The obtained materials were characterized in terms of their other functional properties, and the results are presented in Table 5.

All of the open-cell PUR foams obtained from modified fruit seed oils exhibited comparable apparent densities, ranging from 10.4 to 11.1 kg/m^3^, which are typical values for low-density open-cell polyurethane foams. The slightly higher density observed for the PU_PG foam is not large in absolute terms but may still influence the thermal insulation performance. In materials with such low apparent densities, even small differences in cell structure and solid phase content can noticeably affect the thermal conductivity coefficient.

The thermal conductivity (λ) values of the foams ranged from 40.1 to 44.0 mW/m·K, which are slightly higher than those commonly reported for commercial open-cell PUR foams (36–38 mW/m·K [37]). The highest λ value obtained for PU_PG correlates with its slightly increased apparent density and likely reflects a more compact cellular structure resulting from the higher viscosity and molecular weight of the corresponding biopolyol. Although the differences are minor, they suggest that in low-density foams, even subtle variations in morphology and density can contribute to measurable changes in thermal conductivity.

The compressive strength values (22.9–25.8 kPa in the parallel and 11.8–15.0 kPa in the perpendicular direction) remained within the range typical for commercial open-cell PUR foams, confirming that the mechanical performance of the obtained bio-based foams is comparable to conventional fossil-derived materials. These results demonstrate that foams synthesized from fruit seed oil-based biopolyols can serve as sustainable alternatives for thermal insulation applications.

## 4. Conclusions

A comprehensive characterization of five fruit seed oils constituting a potential source of raw materials for the synthesis of biopolyols was performed. Pomegranate seed oil was found to exhibit the highest viscosity (216 mPa·s) as compared to cherry seed oil (63 mPa·s), grape seed oil (49 mPa·s), blackcurrant seed oil (46 mPa·s) and watermelon seed oil (53 mPa·s). Five types of biopolyols derived from the fruit seed oils were used as renewable raw materials for the preparation of open-cell polyurethane foams with thermal insulating properties. The transesterification of fruit seed oils using triethanolamine was carried out to obtain reactive hydroxyl derivatives enriched with nitrogen, which contributes to the higher reactivity of the biopolyols. The biopolyols were found to have properties suitable for the production of open-cell polyurethane foams. It has been shown that the viscosity of the oils significantly affects the properties of the foams derived from them. The biopolyol obtained from pomegranate seed oil was characterized by the highest viscosity, which consequently influenced the course of the foaming process and had an impact on the value of the thermal conductivity coefficient. Based on the analysis of the foaming process, a different course of the cross-linking process was determined in comparison to the other biopolyols. However, the foaming process can be adjusted with the use of appropriate catalytic systems. Our research demonstrates that achieving the goals set by the European Union is realistic given the possibility of using various raw materials of natural origin, including waste, in the synthesis of biopolymers. Future studies should focus on optimizing thermal conductivity and adjusting catalytic formulations to achieve better control over the foaming process and cellular structure.

## Figures and Tables

**Table 1:** Formulations of polyol premixes (in grams).

Component	PU_BC	PU_CH	PU_GR	PU_PG	PU_WM
P_BC, g	100	0	0	0	0
P_CH, g	0	100	0	0	0
P_GR, g	0	0	100	0	0
P_PG, g	0	0	0	100	0
P_WM, g	0	0	0	0	100
Polycat^®^ 15, g	1	1	1	1	1
Polycat^®^ 140, g	5	5	5	5	5
Kosmos^®^ 19, g	1	1	1	1	1
TEGOSTAB^®^ B 8526, g	3	3	3	3	3
ORTEGOL^®^ 500, g	0.5	0.5	0.5	0.5	0.5
TCPP, g	30	30	30	30	30
Water, g	15	15	15	15	15

**Table 2:** Comparison of fatty acid profiles of fruit seed oils.

Designation	Content, %				
	O_BC	O_CH	O_GR	O_PG	O_WM
C8:0	0.01 ± 0.00		0.01 ± 0.00		
C10:0	0.01 ± 0.00	0.01 ± 0.00	0.01 ± 0.00		
C12:0	0.02 ± 0.00	0.01 ± 0.00	0.01 ± 0.00		
C14:0	0.09 ± 0.00	0.06 ± 0.01	0.06 ± 0.00	0.09 ± 0.01	0.05 ± 0.00
C15:0	0.03 ± 0.00	0.03 ± 0.00	0.01 ± 0.00	0.10 ± 0.01	0.05 ± 0.00
C16:0	7.01 ± 0.02	9.93 ± 0.06	9.17 ± 0.06	3.24 ± 0.02	14.67 ± 0.01
C16:1	0.12 ± 0.00	0.45 ± 0.00	0.11 ± 0.00	0.22 ± 0.01	0.11 ± 0.00
C17:0	0.07 ± 0.00	0.11 ± 0.00	0.07 ± 0.00	0.33 ± 0.01	0.08 ± 0.00
C17:1	0.06 ± 0.00	0.13 ± 0.00	0.03 ± 0.00	0.00 ± 0.00	0.01 ± 0.00
C18:0	1.80 ± 0.04	2.92 ± 0.02	4.91 ± 0.07	8.68 ± 0.05	13.13 ± 0.02
C18:1	5.57 ± 0.03	22.45 ± 0.33	6.82 ± 0.08	9.90 ± 0.03	12.87 ± 0.00
C18:2	53.53 ± 0.08	59.25 ± 0.28	76.90 ± 0.12	48.19 ± 0.10	57.44 ± 0.04
C18:3n-3	15.30 ± 0.05	0.45 ± 0.01	1.01 ± 0.01	8.51 ± 0.05	0.51 ± 0.00
C18:3n-6	13.51 ± 0.01		0.02 ± 0.00	0.24 ± 0.01	0.09 ± 0.00
C20:0	0.25 ± 0.00	1.59 ± 0.02	0.23 ± 0.02	2.88 ± 0.01	0.48 ± 0.03
C20:1	1.33 ± 0.01	0.74 ± 0.01	0.24 ± 0.01	3.76 ± 0.01	0.17 ± 0.00
C20:2	0.27 ± 0.00	0.10 ± 0.00	0.05 ± 0.00	0.90 ± 0.01	0.03 ± 0.00
C20:3n-3	0.05 ± 0.00	0.03 ± 0.00		1.44 ± 0.01	
C20:3n-6				1.24 ± 0.01	
C20:4	0.02 ± 0.00	0.03 ± 0.00			
C20:5	0.07 ± 0.00	0.35 ± 0.02	0.03 ± 0.00	0.00 ± 0.00	0.03 ± 0.00
C21:0	0.02 ± 0.00	0.04 ± 0.00	0.02 ± 0.00		
C22:0	0.19 ± 0.01	0.88 ± 0.01	0.06 ± 0.00	7.88 ± 0.04	0.11 ± 0.00
C22:1	0.19 ± 0.01	0.06 ± 0.00	0.10 ± 0.01	0.44 ± 0.01	
C22:2	0.11 ± 0.01	0.07 ± 0.00	0.05 ± 0.01	0.37 ± 0.01	
C22:6	0.06 ± 0.01		0.01 ± 0.00	0.13 ± 0.01	
C24:0	0.17 ± 0.00	0.28 ± 0.01	0.03 ± 0.00	0.63 ± 0.01	0.14 ± 0.00
C24:1	0.15 ± 0.00	0.04 ± 0.00	0.01 ± 0.01	0.84 ± 0.01	0.06 ± 0.00
SFA	9.67	15.86	14.59	23.83	28.69
MUFA	7.42	23.87	7.31	15.16	13.22
PUFA	82.91	60.27	78.10	61.01	58.09

**Table 3:** Properties of oils used for preparation of biopolyols.

Type of Oil	Mn, g/mol	Mw, g/mol	D, -	d, g/cm^3^	ƞ, mPa·s	Iv, gI_2_/100 g
O_BR	770	810	1.05	0.93	45.7	142.9
O_CH	820	850	1.04	0.92	62.9	137.5
O_GR	850	860	1.01	0.92	48.6	133.3
O_PG	880	890	1.01	0.94	216	102.7
O_WM	870	870	1.01	0.92	52.5	118.3

**Table 4:** Properties of biopolyols.

Type of Oil	Mn, g/mol	Mw, g/mol	D, -	f, -	d, g/cm^3^	ƞ, mPa·s	OHv, mgKOH/g	%H_2_O, %
O_BR	270	470	1.71	1.9	0.98	180	385	0.37
O_CH	270	480	1.77	1.8	0.98	210	375	0.33
O_GR	270	470	1.72	1.7	0.98	150	360	0.31
O_PG	340	620	1.84	2.2	0.99	530	370	0.24
O_WM	280	480	1.71	1.9	0.98	190	375	0.33

**Table 5:** Properties of open-cell polyurethane foams.

Properties	PU_BR	PU_CH	PU_GR	PU_PG	PU_WM
d, kg/m^3^	10.7 ± 0.4	10.6 ± 0.4	10.4 ± 0.5	11.1 ± 0.3	10.5 ± 0.5
CC, %	<5%	<5%	<5%	<5%	<5%
λ, mW/mK	40.2 ± 0.1	41.4 ± 0.7	40.7 ± 0.3	44.0 ± 0.1	40.1 ± 0.2
σ pa, kPa	23.2 ± 2.4	24.3 ± 2.7	25.8 ± 2.2	22.9 ± 1.5	24.0 ± 2.7
σ pe, kPa	12.3 ± 1.5	14. 3 ± 2.9	15.0 ± 1.4	13.8 ± 1.5	11.8 ± 3.5

d—apparent density; CC—content of closed cells, λ—coefficient of thermal conductivity; σ pa—compressive strength at 10% strain measured in a direction parallel to the growth direction; σ pe—compressive strength at 10% strain measured in a direction perpendicular to the growth direction.

## Data Availability

The original contributions presented in this study are included in the article. Further inquiries can be directed to the corresponding author.
